# Thin Film Piezoelectric Nanogenerator Based on (100)-Oriented Nanocrystalline AlN Grown by Pulsed Laser Deposition at Room Temperature

**DOI:** 10.3390/mi14010099

**Published:** 2022-12-30

**Authors:** Wei Li, Yunqi Cao, Nelson Sepúlveda

**Affiliations:** 1 Department of Mechanical Engineering, University of Vermont, 33 Colchester Ave., Burlington, VT 05405, USA; 2 College of Control Science and Engineering, Zhejiang University, 38 Zheda Rd., Hangzhou 310027, China; 3 Department of Electrical and Computer Engineering, Michigan State University, 428 S. Shaw Lane, East Lansing, MI 48824, USA

**Keywords:** piezoelectric nanogenerator, aluminum nitride, energy harvesting, pulsed laser deposition, piezoelectricity, piezoresponse force microscopy, wearable device

## Abstract

In wearable or implantable biomedical devices that typically rely on battery power for diagnostics or operation, the development of flexible piezoelectric nanogenerators (NGs) that enable mechanical-to-electrical energy harvesting is finding promising applications. Here, we present the construction of a flexible piezoelectric nanogenerator using a thin film of room temperature deposited nanocrystalline aluminium nitride (AlN). On a thin layer of aluminium (Al), the AlN thin film was grown using pulsed laser deposition (PLD). The room temperature grown AlN film was composed of crystalline columnar grains oriented in the (100)-direction, as revealed in images from transmission electron microscopy (TEM) and X-ray diffraction (XRD). Fundamental characterization of the AlN thin film by piezoresponse force microscopy (PFM) indicated that its electro-mechanical energy conversion metrics were comparable to those of c-axis oriented AlN and zinc oxide (ZnO) thin films. Additionally, the AlN-based flexible piezoelectric NG was encapsulated in polyimide to further strengthen its mechanical robustness and protect it from some corrosive chemicals.

## 1. Introduction

Flexible thin film-based NGs have been demonstrated to have a positive impact on energy harvesting applications, particularly due to their ability to scavenge biomechanical energy from the human body. They are capable of converting very small amounts of in vivo biomechanical energy from a variety of sources, such as diaphragm movement, muscle relaxation and contraction, heartbeat, and blood flow [1,2,3,4,5,6,7,8,9]. Tactile sensors, pacemakers, artificial skin, heart rate monitors, neural stimulators, and implantable cardioverter-defibrillators are examples of bioelectronic devices that employ NGs to offer continuous diagnosis and therapy [10,11,12,13,14,15,16,17].

Lead zirconate titanate (PZT) and ZnO are currently the two inorganic piezoelectric materials for NG fabrication that have attracted the most attention. On the one hand, due to its high piezoelectric coefficient, PZT has long been the preferred piezoelectric material for mechanical energy harvesting [18,19,20,21,22]. It has been extensively utilized in precision motion systems [23,24,25] and microrobotic systems [26,27,28]. PZT typically needs to be baked and annealed at high temperatures (such as above 600 °C), and the presence of lead raises concerns about its long-term in vivo operation, possibly preventing its use in a variety of wearable or biocompatible technologies [29]. On the other hand, ZnO has emerged as a well-researched and widely-used inorganic piezoelectric material for wearable NGs due to its biocompatibility to some extent [29,30,31,32,33,34,35]. In contrast to inorganic ferroelectric materials, ZnO does not require additional electric–thermal poling and could be regarded as a more environmentally friendly piezoelectric material than PZT [36,37].

AlN is a piezoelectric material that is compatible with CMOS and MEMS. Typically, magnetron sputtering is used to deposit (002)-oriented AlN thin films [38,39]. As a different inorganic piezoelectric material, AlN has not been investigated as much as PZT and ZnO in wearable bioimplants. AlN can be grown as thin films and has the same merits as ZnO that were previously mentioned. Moreover, whereas Zn is a fast diffusing ion and may be problematic during the integration of ZnO films in monolithically integrated devices [40,41], AlN is more stable and is compatible with standard silicon technologies [42]. AlN is also biocompatible and features lower mass density, higher electrical resistivity, wider band gap, and resistance to harsh environments [43,44,45,46,47,48]. However, the high temperatures typically required to deposit highly oriented AlN thin films in the c-axis may limit their use in flexible electronics. The research reported here shows the development and characterization of a flexible and biocompatible NG based on (100)-oriented nanocrystalline AlN thin film deposited at room temperature using the PLD technique. As polymers are frequently employed as substrates, building blocks, protective layers, etc. for flexible electronics, the room temperature deposition environment might make it possible to develop AlN-based flexible electronics using a variety of polymers that typically cannot withstand high temperatures.

## 2. Experimental Section and Discussion

### 2.1. Fabrication of Flexible AlN Piezoelectric Nanogenerators

**Figure 1:**
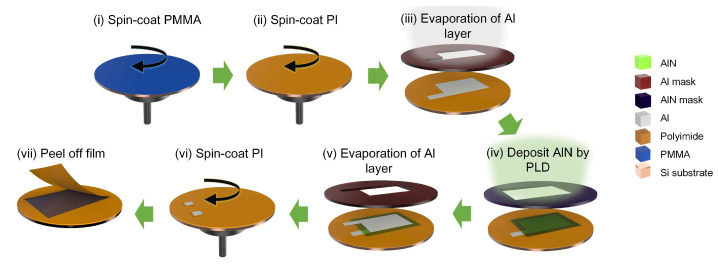
Fabrication procedure of the developed AlN-PNG.

**Figure 2:**
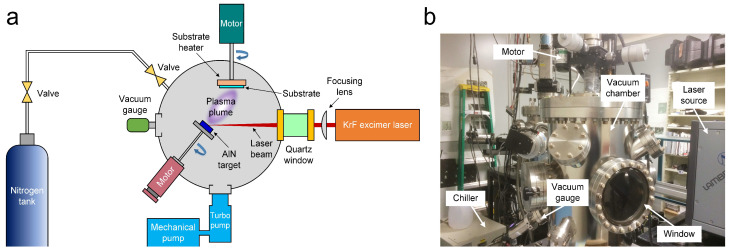
(**a**) Schematic illustration and (**b**) photography of the growth process of AlN thin film by PLD.

The fabrication procedure of the proposed flexible AlN piezoelectric nanogenerator (AlN-PNG) is shown in Figure 1. Initially, a 100-nm-thick polymethyl methacrylate (PMMA; 200336, Sigma-Aldrich, St. Louis, MO, USA) layer was spin-coated onto a 2-inch single-side polished silicon wafer at a speed of 3000 rpm for 1 min. After 30 min, a polyimide (PI, PI-2525, HD MicroSystem, Parlin, NJ, USA) layer with a thickness of around 13 μm was spun at a speed of 1500 rpm for 1 min. Once PI was cured at a temperature of 200 °C for 120 min, a thin layer of Al with a thickness of around 120 nm was evaporated using a shadow mask to define the pattern of the bottom electrode. Al was evaporated in a vacuum chamber (base pressure of 7 mTorr). Next, PLD was used to deposit an 800 nm AlN thin film in a different vacuum chamber (Figure 2). The vacuum chamber was continuously evacuated to a base pressure of less than 8 × 10^−6^ Torr. A stoichiometric hot compressed AlN target (99.8% pure, Kurt J. Lesker, Jefferson Hills, PA, USA) was ablated using a pulsed KrF excimer laser (LPX 200, Lambda Physik, Göttingen, Germany) at a wavelength of 248 nm while it was being rotated by an external motor during the PLD process. The laser’s output energy and its repetition rate were set to 300 mJ and 10 Hz, respectively. An embedded thermocouple in the substrate holder was used to monitor the sample’s temperature. The substrate, which was mounted 4 cm away, received the ablated plume that had been ejected onto it at a 45° angle when the AlN target was struck by a high energy pulsed laser beam. Using a different shadow mask than the one used for the first Al layer, another layer of Al thin film measuring approximately 120 nm was evaporated on top of the AlN thin film. The top and bottom electrodes, which were deposited using two separate Al evaporation procedures, were then electrically isolated and partially exposed. Then, a portion of each Al electrode was covered with polytetrafluoroethylene (PTFE) tape. The second layer of PI (13 μm) was spun at 1500 rpm for one minute after the electrode connections had been covered. After spinning, the PTFE tapes were peeled off, exposing the contact pads at the device’s bottom and top electrodes. Afterward, the PI was cured at 200 °C. The final step of the fabrication procedure was to submerge the wafer in acetone for two hours to dissolve the PMMA layer underneath; this process will separate the thin film device from the wafer so that it can be retrieved eventually.

**Figure 3:**
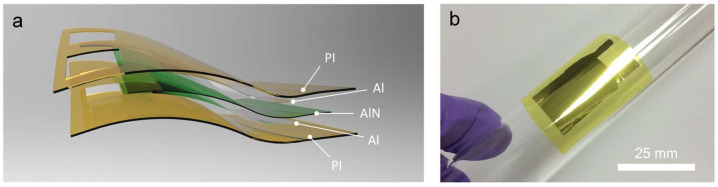
(**a**) Schematic illustration of AlN-PNG and (**b**) optical image of a bent AlN-PNG attached to a glass tube.

Figure 3a displays a schematic illustration of the fabricated AlN-PNG in exploded view. The thin AlN film that PLD deposited at room temperature separated the top and bottom Al layers that served as electrodes. AlN has a high electric resistance (10^11^–10^13^ Ω·cm) and a wide band gap (6.2 ± 0.1 eV) [49]. Previous studies had employed one or more AlN interlayers to build high potential barriers and prevent short circuits between ZnO nanowires (NWs) and the device’s upper electrode [36]. Therefore, compared with other inorganic thin film-based NGs, the developed device did not require an extra insulating layer between the electrodes and the piezoelectric layers. A high potential barrier was provided by the high resistivity of the AlN for preventing undesired current leakage across the electrodes. In order to reduce the risk of performance failure or an immune response, two PI films served as the protective layers that encapsulated the device and made it potentially suitable for implantable biomedical applications [50]. The PI films aim to increase the device’s mechanical robustness without compromising its flexibility and also isolate it from bodily fluids and tissue. An optical image of the bent AlN-PNG attached to a glass tube is shown in Figure 3b, demonstrating its thin film properties and flexibility. It is worth mentioning that, other than PI, additional materials can also be used as the protective layers. For example, hydrogel have been proven recently as a good candidate to serve as both a flexible matrix and conductive building blocks [51,52,53].

### 2.2. Characterization of AlN Thin Film

**Figure 4:**
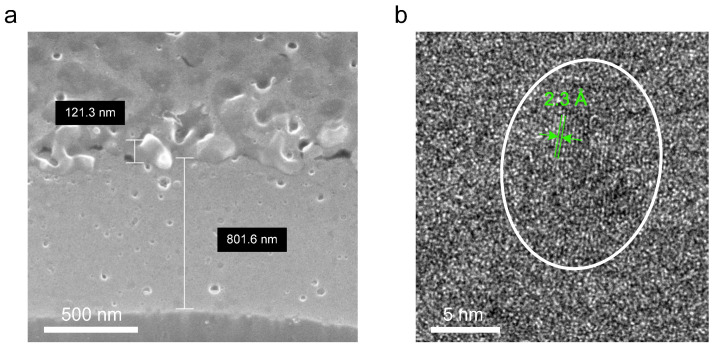
(**a**) FE-SEM image of the PLD-deposited AlN thin film covered with thermally evaporated Al as the electrode. (**b**) TEM image of the PLD-deposited AlN thin film.

**Figure 5:**
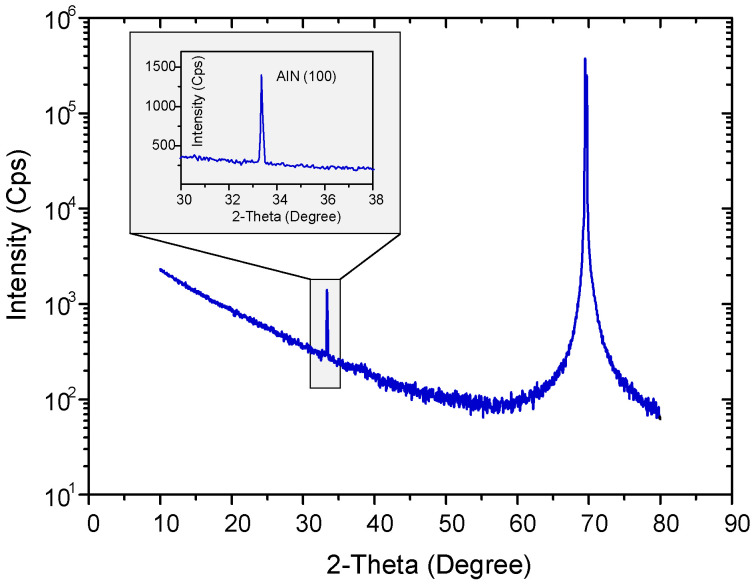
XRD spectrum of the AlN thin film grown by PLD.

Using a field-emission scanning electron microscope (FE-SEM, Carl Zeiss AURIGA, Oberkochen, Germany), a cross-sectional image of the AlN that was deposited by PLD at room temperature and covered with thermally evaporated Al as the electrode was obtained. As can be seen in Figure 4a, the thicknesses of AlN and Al thin films are around 800 nm and 120 nm, respectively. An analysis using TEM (JEOL 2200FS, Tokyo, Japan) was done to observe the AlN crystallographic properties. A TEM image of an around 80-nm-thick AlN film deposited on the TEM grid under the same conditions as AlN-PNG is shown in Figure 4b. In the TEM image, columnar nanocrystalline AlN with a parallel orientation with respect to the substrate is visible. By using an XRD (D5000, Siemens, Berlin, Germany) 2θ scan with Cu Kα_1_ radiation, the crystal structure and crystallinity of the AlN thin film were further characterized. There were no other peaks visible besides the diffraction peak shown in Figure 5 at the angle corresponding to the AlN (100) plane.

### 2.3. Piezoresponse Force Microscopy of AlN Thin Film

**Figure 6:**
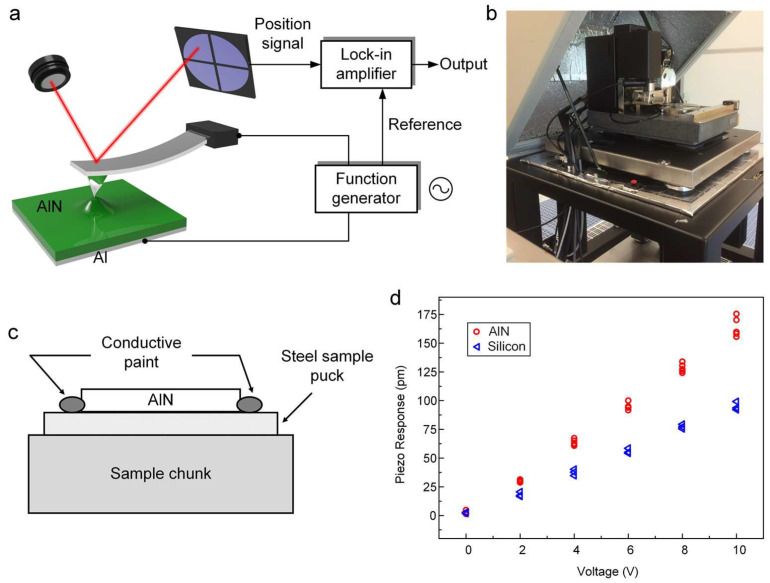
(**a**) Schematic illustration and (**b**) photography of the PFM measurement setup; (**c**) schematic illustration of the sample being prepared on the chunk; (**d**) relationship between piezoresponse amplitude and modulating AC bias voltage amplitude obtained from the PFM measurement.

In order to characterize the piezoelectric property of AlN-PNG, we used a PFM instrument (NanoMan AFM, Bruker, Santa Barbara, CA, USA) to measure the effective longitudinal piezoelectric constant *d*_33_ of the AlN thin film. The PFM technique is a useful method for examining ferroelectric and piezoelectric phenomena [54,55]. The experimental strategy is based on the detection of local sample vibrations caused by an AC signal applied between the conductive tip and the bottom electrode of the sample. The schematic illustration and the photography of the PFM measurement setup are shown in Figure 6a,b, respectively. A layer of Al film was initially deposited on a silicon substrate by evaporation for sample preparation. Then, by means of PLD, an AlN film was deposited onto the Al film. Both the Al film and the AlN film are deposited under the same AlN-PNG fabrication process. The sample was mounted on an electrically conductive puck, and the Al film and puck were electrically connected by applying silver paint, as shown in Figure 6c. In this case, an AlN film of the sample was in contact with a platinum–iridium coated conductive tip (radius of 25 nm, Bruker CONTV-PT, Santa Barbara, CA, USA), and an AC bias voltage was applied to the conductive puck to produce mechanical vibrations. The relationship between applied voltage and displacement was extracted from these vibrations through the demodulation process known as lock-in. The magnitude of the AC bias voltage applied to the PFM tip ranged from 0 to 10 V. The frequency was set at 2 kHz to prevent mechanical oscillation of the tip at its resonant frequency (13 kHz).

It is worth noting that the electrostatic forces and the electromechanical response of the surface may have an impact on the measured signal. Because of this, the measured displacement was not solely a result of the piezoelectric response. Here, we also measured a bare silicon sample as a benchmark in order to eliminate additional displacement sources and isolate the piezoelectric behavior of AlN thin film. The measured amplitude was taken into consideration as the background response because silicon does not exhibit the inverse piezoelectric effect. Figure 6d shows the relationship between piezoresponse amplitude and modulating AC bias voltage amplitude obtained from the PFM measurement. The effect of the background must be taken into consideration when determining the real relationship between the piezoresponse amplitude and the modulation voltage amplitude. The substrate and AlN film were tightly clamped together in the tested structure, which constrained the film’s in-plane contraction and expansion. This type of structure’s measured piezoelectric coefficient can be regarded as an effective piezoelectric coefficient $d_{33}^{eff}$. The calculated effective piezoelectric coefficient for the PLD deposited AlN thin film is 5.9 pm/V. Through the mechanical compliance of the piezoelectric film (*S*_11_, *S*_12_, and *S*_13_) and the transverse piezoelectric coefficient *d*_31_, it has been derived that $d_{33}^{eff}$ is related to the piezoelectric coefficient *d*_33_ in the following manner [56]:

(1) $$d_{33}^{eff}≅d_{33}-\frac{2S_{13}}{S_{11}+S_{12}}d_{31}$$

where the elastic compliance values of AlN, *S*_11_, *S*_12_, and *S*_13_ are taken from Wright [57] as 3 × 10^−12^ m^2^/N, 8 × 10^−12^ m^2^/N, and 8 × 10^−12^ m^2^/N, respectively; and *d*_31_ can be estimated as $d_{33}^{eff}$/2 [58]. Using Equation (1), the unclamped value *d*_33_ of the (100)-oriented AlN thin film used for the reported NG device was calculated to be 10.2 pm/V. The measured values of the effective piezoelectric coefficient *d*_33_ of thin films made of AlN and ZnO are shown in Table 1. As can be seen, the (100)-oriented AlN thin film in this study, which was deposited by PLD at room temperature, had a *d*_33_ piezoelectric constant that was on par with findings for (002)-oriented AlN thin films [58,59,60,61] and was also comparable to that of (002)-oriented ZnO thin films [62,63,64].

### 2.4. Energy Conversion of AlN-PNG

**Figure 7:**
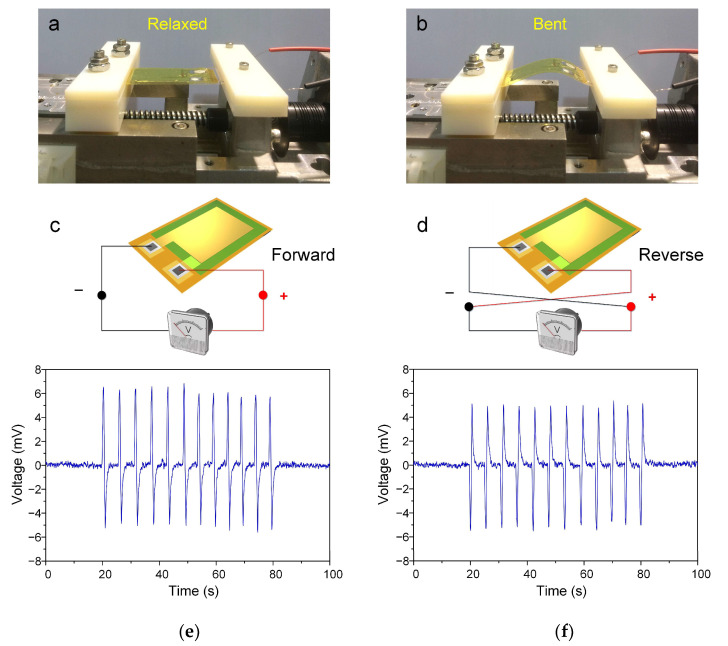
Photographs of AlN-PNG in its (**a**) original state and (**b**) bent state by the linear motion stage; schematic illustration of the open-circuit voltage measurement in (**c**) forward and (**d**) reverse connections; generated voltage from AlN-PNG under periodic bending in (**e**) forward and (**f**) reverse connections.

We measured the open-circuit voltage while the AlN-PNG was being repeatedly subjected to strain inputs in order to validate its performance. This approach has frequently been used to test NGs [3,6,7,66]. A low-noise nanovoltmeter (Keithley 2182A, Cleveland, OH, USA) was used to measure the open-circuit voltage produced by AlN-PNG. By employing a linear motion stage, the AlN-PNG was repeatedly deformed and then restored during the measurement in a cyclic stretching–releasing agitation at a frequency of 0.35 Hz, as depicted in Figure 7a,b, respectively. Switching polarity tests were also conducted, as can be seen in Figure 7c,d, to confirm that the measured signals were not artifacts brought on by the measurement equipment and that the measured output voltage was the result of the piezoelectric effect of the AlN thin film. In Figure 7e,f, the open-circuit voltages obtained from AlN-PNG when they were bent and then released at regular intervals are shown. It is evident that during the continuous cycle of bending and releasing motions, a number of positive and negative pulses were produced. As can be seen, the developed AlN-PNG can generate an open-circuit peak-to-peak voltage of approximately 10 mV. Repeating the experiments three months later allowed for a durability test, and the results demonstrated that the AlN-PNG can still produce the same amount of voltage as when it was initially fabricated. Since the developed device falls under the category of a piezoelectric nanogenerator, it is expected that either increasing the device’s surface area or stacking AlN thin films to create a multi-layer structure will effectively enhance the device’s performance.

## 3. Conclusions

In this work, we have developed a lightweight, flexible, and biocompatible NG based on AlN thin film. The growth of (100)-oriented AlN thin films in a vacuum (i.e., without any background gas) at room temperature was accomplished through the PLD. The XRD measurement revealed the (100)-orientation of the grown AlN thin film. Additionally, PFM testing demonstrated that the thin film of (100)-oriented AlN exhibits a strong piezoelectric response, with a piezoelectric coefficient comparable to that of ZnO and c-axis AlN. Based on the grown AlN thin film, an encapsulated thin film nanogenerator AlN-PNG has been developed. Periodic bending and releasing motion experiments proved the electrical output of the developed AlN-PNG. This work may provide a way towards fabricating inorganic and thin-film structured self-powered electronics and biomedical devices at room temperature.

## Figures and Tables

**Table 1:** Measured effective piezoelectric coefficient $d_{33}^{eff}$ of AlN and ZnO thin films.

Material	$d_{33}^{eff}$(pm/V)	Literature
AlN (002)	4.15	Tonisch et al. [58]
	6.8	Reusch et al. [59]
	2.8–5.2	Martin et al. [60]
	4.6–5.2	Mortet et al. [61]
ZnO (002)	8.14	Li et al. [62]
	13	Christman et al. [63]
	5.9	Safari et al. [64]
AlN (100)	2.3–3.8	Cibert et al. [65]
	5.9	This work

## Data Availability

Not applicable.
